# Simulation of Intergranular Ductile Cracking in *β* Titanium Alloys Based on a Micro-Mechanical Damage Model

**DOI:** 10.3390/ma10111250

**Published:** 2017-10-30

**Authors:** Huan Li, Jinshan Li, Bin Tang, Jiangkun Fan, Huang Yuan

**Affiliations:** 1 State Key Laboratory of Solidification Processing, Northwestern Polytechnical University, Xi’an 710072, China; ljsh@nwpu.edu.cn (J.L.); toby@nwpu.edu.cn (B.T.); jkfan@nwpu.edu.cn (J.F.); 2 School of Mechanics, Civil Engineering and Architecture, Northwestern Polytechnical University, Xi’an 710129, China; 3 School of Aerospace Engineering, Tsinghua University, Beijing 100081, China; yuan.huang@tsinghua.edu.cn

**Keywords:** intergranular crack propagation, *β* titanium alloys, micro-mechanical damage model, ductile fracture, fracture toughness

## Abstract

The intergranular crack propagation of the lamellar structure β titanium alloys is investigated by using a modified Gurson-type damage model. The representative microstructure of the lamellar alloy, which consists of the soft α phase layer surrounding the hard grain interiors, is generated based on an advanced Voronoi algorithm. Both the normal fracture due to void growth and the shear fracture associated with void shearing are considered for the grain boundary α layer. The individual phase properties are determined according to the experimental nanoindentation result and the macroscopic stress–strain curve from a uni-axial tensile test. The effects of the strain hardening exponent of the grain interiors and the void shearing mechanism of the grain boundary α layer on fracture toughness and the intergranular crack growth behavior are emphatically studied. The computational predictions indicate that fracture toughness can be increased with increasing the strain hardening ability of the grain interiors and void shearing can be deleterious to fracture toughness. Based on the current simulation technique, qualitative understanding of relationships between the individual phase features and the fracture toughness of the lamellar alloys can be obtained, which provides useful suggestions to the heat treatment process of the β titanium alloys.

## 1. Introduction

**Figure 1:**
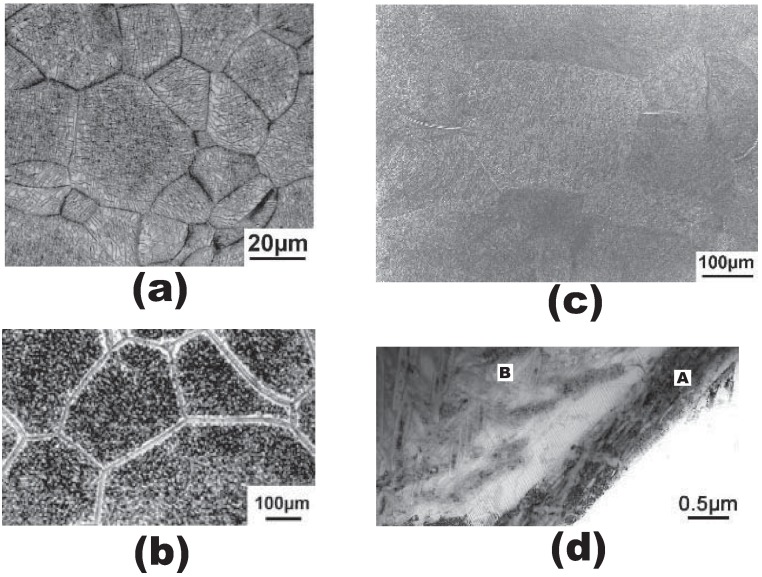
Continuous α layer at grain boundaries in some β titanium alloys: (**a**) β 21S; (**b**) Ti-10V-2Fe-3Al; and (**c**) Ti-5Al-5V-5Mo-3Cr (Ti-5553); (**d**) TEM photograph of continuous α layer (A) and matrix α plate structure (B) in Ti-5553 corresponding to (**c**) (from [1], by permission of © Springer Science + Business Media).

Titanium alloys are commonly used in aerospace engineering due to their high strength, low density, good mechanical properties and excellent heat and corrosion resistance. As the breakthrough product among α/β titanium alloys, the Ti-6Al-4V alloy occupies the most part of the market of aerospace components [1]. In recent years, the β titanium alloys have emerged to replace Ti-6Al-4V in some of aerospace components owing to their excellent comprehensive performance [1,2]. According to the morphology and the distribution of the α phase associated with different heat treatment procedures, there are two commonly used microstructures of the β titanium alloys, namely fully lamellar and bimodal, respectively [3]. The literature focussed on the morphology of the α phase showed that the preparation route of the high ultimate tensile strength for the lamellar alloy is more convenient than that for the bimodal alloy [4]. A typical processing sequence for the lamellar β alloys contains a series of four steps, namely the homogenization (step I), the deformation (step II), the recrystallization (step III) and the annealing plus the aging treatment (step IV), where the first three steps are operated in the β phase field and the final step is normally done in the α + β phase field [1]. After the heat treatment, the representative structure of the lamellar β alloy consists of the continuous thin α phase layer surrounding the hard grain interior, which is composed of the nanoscale α phase laths inside the prior β grain [1,3,4], as shown in Figure 1. Due to the preferential plastic deformation along the continuous α layer and the grain boundary regions are softer than the precipitation hardened grain interiors [1], intergranular ductile fracture is easy to happen [3]. Since the fracture toughness of the β titanium alloys is highly related to intergranular fracture [1,3] and the fracture toughness is one of the key design criteria of the failure-resistant components in aerospace engineering, it is necessary to investigate the intergranular crack propagation behavior of the lamellar β titanium alloys. 

Prior efforts at modeling intergranular fracture of polycrystalline metals have shown that ductility and fracture toughness depends on the material response of the grain interiors, the intergranular failure properties of the grain boundary regions, the distribution of the grain size and orientation [5,6,7,8,9,10,11,12]. One common approach is to simulate the intergranular separation of grain boundaries according to the cohesive interface models [8,9,10,11,12] and the stress–strain behavior of the grain interiors can be described by using the anisotropic elastic theory [11,12], the isotropic Von Mises plasticity [8,9] or the crystal plasticity theory [8,9,10] depending on whether the effects of the plasticity and the orientation of grains are considered. Although the cohesive interface models are helpful to understand the failure mechanisms associated with low ductility or fracture toughness at meso-scale, they are more suitable in simulating the brittle intergranular fracture [8,10] or the cleavage fracture [12,13] of polycrystalline materials. In the β titanium alloys, the intergranular crack initiation sites are commonly considered to be caused by the difference in elastic-plastic deformation between grains [1,3]. Fractographic examinations have also shown that the intergranular fracture of the β titanium alloy occurs on the grain boundary through nucleation of voids, void growth and coalescence [3]. The intergranular failure caused by this micro-mechanism can be well described by the Gurson–Tvergaard–Needleman (GTN) model according to void volume fraction together with the first and the second stress invariants [14,15]. Based on the assumption that damage evolution is determined by cooperation of plastic deformation and high stress triaxiality, the GTN model was extensively used in simulating the gradually degraded yield surface of ductile materials [15,16]; however, few investigations on intergranular ductile fracture have been performed based on this model [7].

For the two-phase titanium alloys including the β alloys, quantitative tilt fractography analysis has been performed to calculate the angular deviation between the loading direction and the initiation facet normal corresponding to intergranular fracture, which reveals that both normal and shear force components of facets are necessary to initiate a crack [3]. Therefore, the deformation process of the grain boundary layer involves the significant variations of stress states; however, it has already be recognized that the GTN model has no ability to simulate fracture at low stress triaxiality [15,17]. In order to encompass a wider scope of stress triaxiality, the original GTN model has been extended by taking into account the effects of the third stress invariant. Some additional parameters are introduced, which set the rate of damage evolution in shear [17,18,19]. These extended models capture the macroscopic experimental observations of various metal alloys in the shear dominated stress states with low stress triaxiality.

Motivated by the issues outlined above, the intergranular ductile fracture of the β titanium alloys will be investigated based on a extended GTN model that considers both the normal fracture due to the internal necking of neighboring voids and the shear fracture associated with the void shearing mechanism. The paper is structured as follows. In Section 2, the extended GTN model in terms of low stress triaxiality is reviewed and the corresponding numerical algorithm is introduced. In Section 3, the finite-element polycrystal model of the lamellar Ti-5Al-5V-5Mo-3Cr (Ti-5553) alloy with the random grain geometry is generated from the Voronoi tessellation at first. Then, the stress–strain responses of both the grain boundary α layer and the grain interiors are determined according to the nanoindentation and the uni-axial tensile tests available in the literature, and finally show the representative simulations of the damage evolution under both tensile and shear loading. In Section 4, a notched Ti-5553 specimen is used to simulate the intergranular crack propagation process. The effects of void shearing of the grain boundary α layer and the elastic-plastic response of the grain interiors on the fracture toughness of Ti-5553 are discussed in detail. Meanwhile, the crack propagation process for two different model parameters are compared to reveal the effects of void shearing. The conclusions are summarized in Section 5.

## 2. Constitutive Modeling

By considering void growth and the internal necking between two adjacent voids, the original GTN model was developed to describe the gradually degraded yield surface depending explicitly upon the void volume fraction *f* of ductile materials [15,16]. The yield function of the model is given below:

(1) $$\Phi ={\left(\frac{\sigma_{eq}}{\sigma_{y}}\right)}^{2}+2q_{1}f^{*}\cosh\left(-\frac{3}{2}q_{2}\frac{\sigma_{m}}{\sigma_{y}}\right)-\left(1+q_{1}^{2}f^{*2}\right),$$

where the constants $q_{1}$ and $q_{2}$ were introduced by Tvergaard and Needleman to improve the predictive ability of the Gurson model [14,20]. $\sigma_{y}\left({\bar{\varepsilon }}^{p}\right)=\sigma_{y0}+R\left({\bar{\varepsilon }}^{p}\right)$ refers to the yield stress of the matrix material, in which $\sigma_{y0}$ is the initial yield stress and *R* is the hardening function associated with the equivalent plastic strain ${\bar{\varepsilon }}^{p}$. $\sigma_{eq}=\frac{3}{2}S:S$ denotes the Von Mises stress and $\sigma_{m}$ is the mean stress. The deviatoric stress $S=\sigma -\sigma_{m}I$, where σ is the macroscopic Cauchy stress tensor and I is the second order identity tensor. The ratio $\sigma_{m}/\sigma_{eq}$ is the stress triaxiality that cooperates with plastic deformation to promote void growth. $f^{*}$ is the equivalent value of the void volume fraction *f*. By introducing the critical void volume fraction $f_{c}$ at the onset of void coalescence, $f^{*}$ is of the bi-linear form as

(2) $$f^{*}=\left\{\begin{array}{ccc} f, & \; & f<f_{c},\\ f_{c}+\kappa \left(f-f_{c}\right), & \; & f_{c}<f<f_{F},\\ f_{u}, & \; & f\geq f_{F}, \end{array}\right.$$

where κ is the acceleration factor for void growth after coalescence. $f_{F}$ indicates the void volume fraction at fracture and $f_{u}=f_{c}+\kappa \left(f_{F}-f_{c}\right)$. By taking into account the void shearing mechanism [17], the evolution equation for *f* is defined by

(3) $$\Delta f=\left(1-f\right)\Delta \epsilon^{p}:I+q_{3}f\omega \left(\sigma \right)\frac{S:\Delta \epsilon^{p}}{\sigma_{eq}}$$

without void nucleation, where $\Delta \epsilon^{p}$ is the macroscopic plastic strain rate tensor. The material constant $q_{3}$ is introduced to represent the damage evolution rate in pure shear state. ω is the shear stress invariant used to quantify the stress state during the damage process. ω is denoted as

(4) $$\omega \left(\sigma \right)=1-{\left(\frac{27\det(S)}{2\sigma_{eq}^{3}}\right)}^{2},$$

where ω lies between 0 and 1, ω=0 for the tensile stress state and ω=1 for the pure shear stress state plus an arbitrary mean stress. The operator det(S) stands for the determinant of the deviatoric stress tensor S. It needs to be emphasized that *f* in Equation (3) can not be considered as the void volume fraction any more in the extended model, it defines the damage counter similar to the damage variable in the continuum damage mechanics theory [15,17]. The second term in Equation (3) introduces the material softening due to void deformation and reorientation. The onset of fracture is predicted when *f* reaches $f_{F}$ and the material is considered to fracture.

The constitutive equation of materials based on Hooke’s law can be written in an incremental form from time *t* to *t* + Δt as

(5) $$\sigma_{t+\Delta t}=2G\epsilon_{t+\Delta t}^{e}+\lambda II:\left(\epsilon_{t+\Delta t}^{e}\right)=2G\left(\epsilon_{t}^{e}+\Delta \epsilon^{e}\right)+\lambda II:\left(\epsilon_{t}^{e}+\Delta \epsilon^{e}\right),$$

where *G* is the shear modulus and λ is the Lame constant. Since the elastic increment strain $\Delta \epsilon^{e}=\Delta \epsilon -\Delta \epsilon^{p}$, Equation (5) can be written in its predictor–corrector form in the framework of the backward Euler method [21] as

(6) $$\sigma_{t+\Delta t}=\sigma^{e}-\left(2G\Delta \epsilon^{p}+\lambda II:\Delta \epsilon^{p}\right),$$

where Δϵ is the total strain increment and $\Delta \epsilon^{p}$ is the plastic strain increment. $\sigma^{e}=2G\left(\epsilon_{t}^{e}+\Delta \epsilon \right)+\lambda II:\left(\epsilon_{t}^{e}+\Delta \epsilon \right)$ denotes the elastic predictor which can be alternatively written as

(7) $$\sigma^{e}=S^{e}+\sigma_{m}^{e}I,$$

where the deviatoric elastic predictor and the predictor of means stress are defined by

(8) $$S^{e}=2Ge=2G\left[\epsilon -\left(\frac{\mathrm{II}:\epsilon }{3}\right)\right],\sigma_{m}^{e}=K\left(\frac{I:\epsilon }{3}\right).$$

The quantity e represents the deviator of strain and *K* is the elastic bulk modulus. The yield function and the flow rule are given by

(9) $$\Phi \left(\sigma_{m},\sigma_{eq},\sigma_{y}\left({\bar{\varepsilon }}^{p}\right),f^{*}\right)=0,$$

(10) $$\Delta \epsilon^{p}=\frac{1}{3}\Delta \varepsilon_{m}I+\Delta \varepsilon_{eq}n_{t+\Delta t},$$

with

(11) $$n=\frac{3}{2}\frac{S}{\sigma_{eq}},\;\Delta \varepsilon_{m}=\Delta \lambda \frac{\partial \Phi }{\partial \sigma_{m}},\;\Delta \varepsilon_{eq}=\Delta \lambda \frac{\partial \Phi }{\partial \sigma_{eq}}.$$

Eliminating the plastic multiplier Δλ in above equations, the following constraint equation is obtained,

(12) $$\Delta \varepsilon_{m}\frac{\partial \Phi }{\partial \sigma_{eq}}-\Delta \varepsilon_{eq}\frac{\partial \Phi }{\partial \sigma_{m}}=0.$$

The stress tensor at time t+Δt can be determined by

(13) $$\sigma_{t+\Delta t}=\sigma_{m_{t+\Delta t}}I+S_{t+\Delta t}=\sigma_{m_{t+\Delta t}}I+\frac{2}{3}\sigma_{{eq}_{t+\Delta t}}n_{t+\Delta t}.$$

The combination of Equations (6) and (10) results in

(14) $$\sigma_{t+\Delta t}=\sigma^{e}-K\Delta \varepsilon_{m}I-2G\Delta \varepsilon_{eq}n_{t+\Delta t}.$$

Since $S^{e}$ and $S_{t+\Delta t}$ are coaxial in the deviatoric stress space according to Equation (14), $n_{t+\Delta t}$ can be determined through the elastic predictor as

(15) $$n_{t+\Delta t}=\frac{3}{2}\frac{S^{e}}{{\sigma_{eq}}^{e}}.$$

Projecting Equation (14) to I and n, then the following equations can be derived in comparison with Equation (13):

(16) $$\sigma_{m_{t+\Delta t}}={\sigma_{m}}^{e}-K\Delta \varepsilon_{m},$$

(17) $$\sigma_{{eq}_{t+\Delta t}}={\sigma_{eq}}^{e}-3G\Delta \varepsilon_{eq}.$$

By assuming that the equivalent plastic work principle is always satisfied, the relationship between the equivalent plastic strain ${\bar{\varepsilon }}^{p}$ of the matrix and the macroscopic plastic strain is given by

(18) $$\left(1-f\right)\sigma_{y}\Delta {\bar{\varepsilon }}^{p}=\sigma :\Delta \epsilon^{p}.$$

Finally, all the corresponding equations for the extended GTN model are summarized as follows:

(19) $$\begin{array}{c} \Phi \left(\sigma_{m},\sigma_{eq},\sigma_{y}\left({\bar{\varepsilon }}^{p}\right),f^{*}\right)=0,\\ \Delta \varepsilon_{m}\frac{\partial \Phi }{\partial \sigma_{eq}}-\Delta \varepsilon_{eq}\frac{\partial \Phi }{\partial \sigma_{m}}=0,\\ \sigma_{m}={\sigma_{m}}^{e}-K\Delta \varepsilon_{m},\\ \sigma_{eq}={\sigma_{eq}}^{e}-3G\Delta \varepsilon_{eq},\\ \Delta \sigma_{y}=\frac{d\sigma_{y}}{d{\bar{\varepsilon }}^{p}}\Delta {\bar{\varepsilon }}^{p}=\frac{d\sigma_{y}}{d{\bar{\varepsilon }}^{p}}\frac{\sigma_{m}\Delta \varepsilon_{m}+\sigma_{eq}\Delta \varepsilon_{eq}}{\left(1-f\right)\sigma_{y}},\\ f=f+\Delta f,\\ \Delta f=\left(1-f\right)\Delta \epsilon^{p}:I+q_{3}f\omega \left(\sigma \right)\frac{S:\Delta \epsilon^{p}}{\sigma_{eq}}\\ =\left(1-f\right)\Delta \varepsilon_{m}+q_{3}f\omega \left(\sigma \right)\Delta \varepsilon_{eq}. \end{array}$$

The above system of nonlinear equations for the unknowns $\sigma_{m}$, $\sigma_{eq}$, $\Delta \varepsilon_{m}$, $\Delta \varepsilon_{eq}$ and *f* are solved by means of the Newton–Raphson method [22]. The integration algorithm of the extended GTN model has been implemented into the commercial code ABAQUS according to the user subroutine UMAT (user-defined material) [23].

## 3. Parameters Determination and Model Validation

### 3.1. Estimation of Individual Phase Properties

The mechanical properties of the β alloys are dominated by the preferential plastic deformation along the continuous α layers at β grain boundaries as indicated by Lütjering et al. in Ref. [1], this phenomenon has been confirmed in some widely used β alloys such as Ti-6246 and Ti-5553 with lamellar structure for aerospace applications [1,3]. In the present study, the intergranular crack propagation behavior is investigated according to Ti-5553. Based on the experimental observations, the typical structure of the lamellar Ti-5553 alloy is composed of the continuous thin α phase layer surrounding the prior β grain, which is embedded by the α phase laths [1,3,4], as displayed in Figure 1c,d. In simulations, it is important to determine the microstresses for each constituent phase during deformation. Since the mechanical behavior of the nanoscale α phase precipitates inside the prior β grain is difficult to obtain, each precipitation hardened grain is treated as a homogeneous and isotropic grain; meanwhile, the grain boundary α phase layer and the hard grain interiors are assumed to follow the elastic-plastic isotropic hardening behavior.

**Figure 2:**
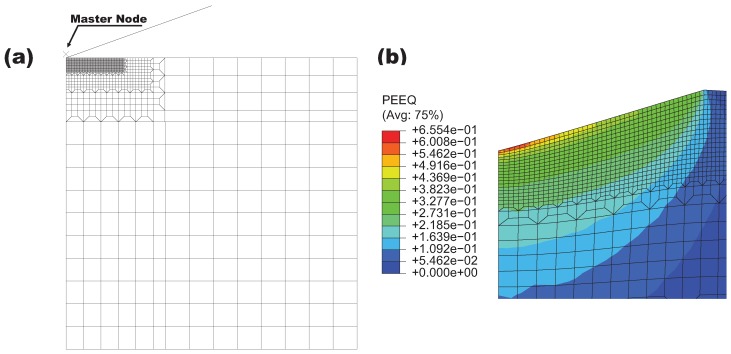
(**a**) axisymmetric finite-element model with a conical indenter for the nanoindentation simulation; (**b**) contour plot of the equivalent plastic strain (PEEQ) within the α phase layer near the tip of the conical indenter after unloading.

According to the existing literature [24,25], the stress–strain curve of α phase can be determined by fitting the nanoindentation load-depth curve. Since the mechanical properties of each individual phase of metal alloys depends on various factors such as chemical composition, microstructure and thermal processing [13,26,27], the reported α phase properties for Ti-5553 are different. For qualitative investigation, a experimental nanoindentation test reported in Ref. [26] is adopted to identify the mechanical properties of the α phase layer. A three-sided Berkovich indenter with a total included angle of 142.3° is used in their tests. Based on several studies [28,29], nanoindentation tests using a conical indenter with a half apex angle of 70.3° yields the same load-depth curve as the Berkovich indenter, thus an axisymmetric finite-element model is developed as shown in Figure 2a. The mesh near the contact region is refined to ensure the accuracy of the simulation results, and the minimum size of four-nodal elements equals 10 nm. In simulations, the vertical movement of the nodes at the bottom boundary is constrained; meanwhile, the horizontal movement of the nodes on the left boundary is constrained due to the symmetry condition. The loading process is performed by applying the downward displacement on the master node of the rigid indenter and the subsequent unloading is achieved by removing the applied displacement of the indenter at the peak reaction force.

**Figure 3:**
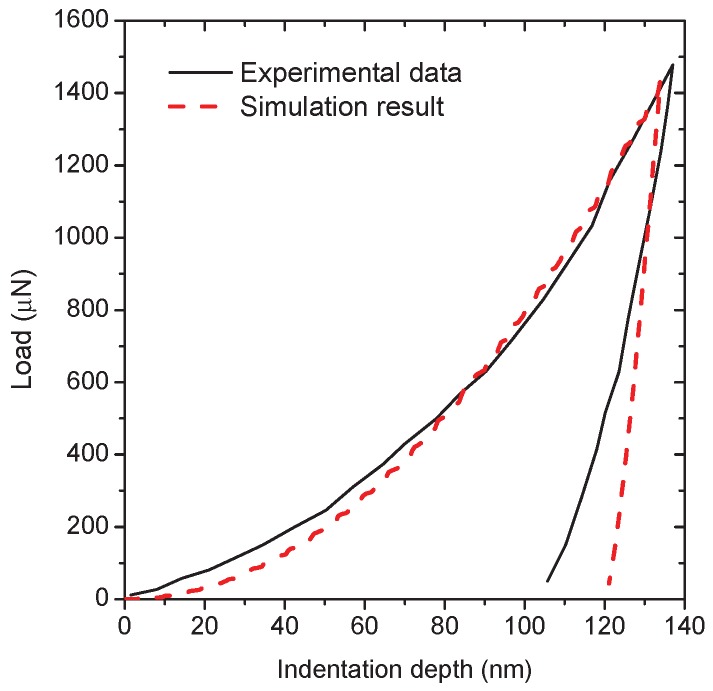
Comparison of numerical and experimental load-depth curves for the α phase layer of the lamellar Ti-5553 alloy.

Because the general elastic parameters found in the open literature for the α phase of the titanium alloys have little difference [26,30], the isotropic elastic parameters identified in Ref. [26] are considered to be the known parameters, namely, the Young’s modulus *E* is 125 GPa and the Poisson’s ratio is 0.33, the nanoindentation simulations are concentrated in determining the plastic properties. After a large number of numerical calculations, the relationship between true stress $\sigma_{t}$ and the true plastic strain ${\varepsilon_{t}}^{p}$ can be well described by the Holomon equation $\sigma_{t}=K_{1}{\left({\varepsilon_{t}}^{p}\right)}^{n_{1}}$ with ${\varepsilon_{t}}^{p}\geq 0.2\%$, where the strength coefficient $K_{1}$ = 889 MPa and the strain hardening exponent $n_{1}$ = 0.027. Figure 2b displays the equivalent plastic strain within the α phase near the tip of the indenter. The comparison between the simulation and the experimental load-depth curve is shown in Figure 3. The two results are matched each other very well at the loading stage, while the deviation exists at the final stage of the unloading part. Since the unloading part is highly sensitive to the Young’s modulus, this numerical result is the best fitting based on the known isotropic elastic parameters and the given indenter-tip geometry. 

In accordance with the popular approach to obtain the material properties of each individual phase [31,32], a experimentally measured stress–strain curve in uni-axial tension from specimens of the lamellar Ti-5553 [27] is used to estimate the material parameters of the grain interiors. The chemical composition of the material is given in Table 1. The heat treatment produces a microstructure composed of refined α precipitates in the prior β grain together with the grain boundary α phase layer, and this microstructure often has the higher tensile strength accompanied by some ductility. The tensile tests show that the ultimate strength is between 1093–1201 MPa, and the fracture strain ranges from 4.7–15.9% [27].

**Figure 4:**
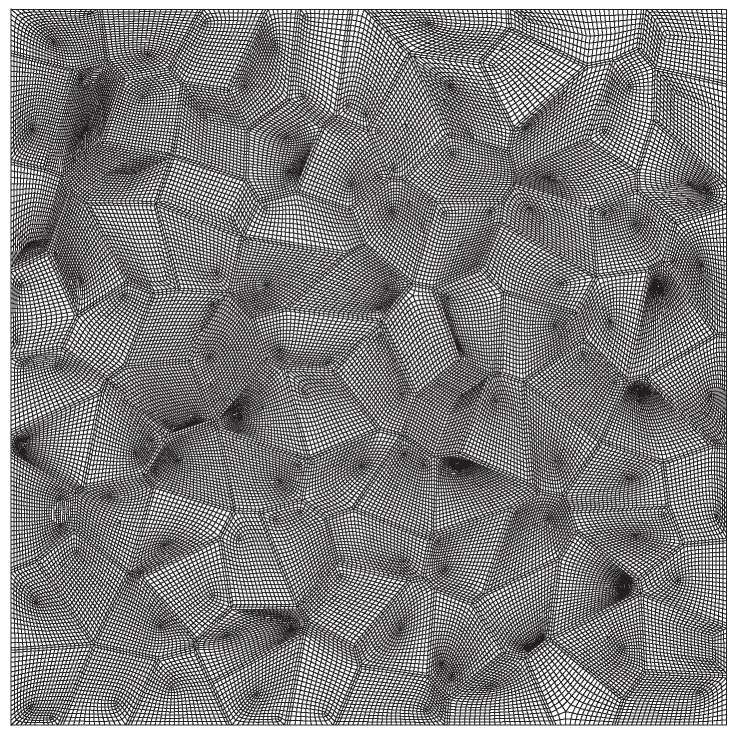
The finite-element model used for the representative volume element (RVE) of the lamellar Ti-5553.

Typically, the grain size of the β titanium alloys including Ti-5553 lies in the range of 25–500 μm, and the ratio of the grain boundary α layer thickness to the grain size ranges from $10^{-2}$–$10^{-4}$ [7]. Because of the need to determine the elastic and plastic parameters of the hard grain interiors, the advanced Voronoi algorithm [33] is adopted to generate the more realistic two-dimensional grain structure of the polycrystal, which is used as the representative volume element (RVE) of the Ti-5553 alloy, as shown in Figure 4. Since the ratio between the average grain size and the thickness of the grain boundary α layer is too small to be generated in the finite-element model, the RVE in simulations is a 600 × 600 μm square consisting of 100 grains, and the thickness of the grain boundary α layer is 4 μm. Refer to the general perspective of crack propagation modeling [7,34], the grain boundary normally has the finite thickness of two finite elements. The RVE captures the main features of the grain structure in the lamellar Ti-5553 as displayed in Figure 1c, thus it can be used to study the interaction between the grain interiors and the grain boundaries reasonably. In order to calculate macrostresses from the finite-element representation of the microstructure, two-dimensional plane strain elements are applied to simulate the material behavior of the Ti-5553 specimen under tension. The grain boundaries in this model are explicitly modeled with finer meshes for simulating intergranular ductile fracture. 

During the simulation of the uni-axial tension, all the nodes at the top edge are given the same displacements in the vertical direction. To avoid the movement of rigid body, one of the top nodes is constrained not to move in the horizontal direction and the rest of the top nodes can freely move in the horizontal direction during deformation. Meanwhile, all the nodes at the bottom edge are constrained not to move in the vertical direction, but can move freely in the horizontal direction. In the post process of modeling, the macroscopic engineering stress is derived by dividing the reaction force of the RVE in the vertical direction with the initial area of 600 $\mu m^{2}$. The engineering strain is derived by dividing the vertical displacement of the top edge with the initial length 600 μm of the RVE.

**Figure 5:**
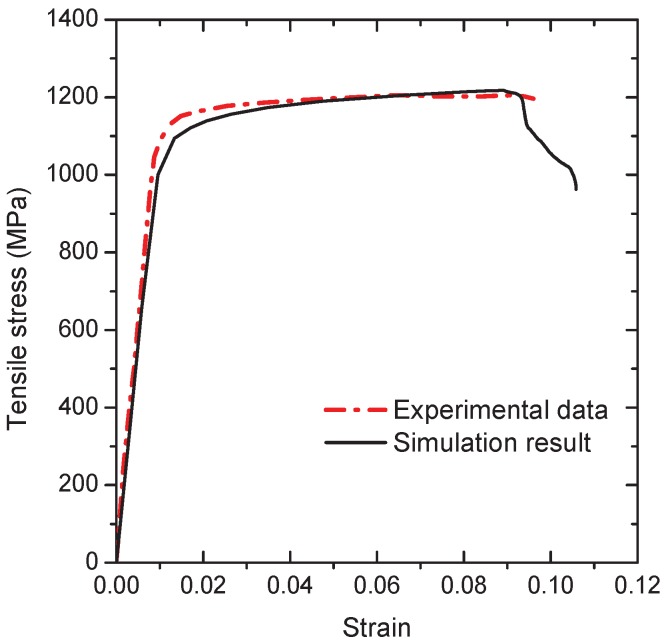
The engineering stress–strain curve of the RVE under uniaxial tension based on the fitted input stress–strain curves for the grain boundary α layer and the grain interiors.

By considering that the material softening happens due to the formation of the intergranular crack, the extended GTN model introduced in Section 2 is adopted to determine the damage evolution along the grain boundaries. Over a large number of numerical testings, the best estimation of the experimental stress–strain curve is given by the following flow curve of the grain interiors, namely,

(20) $$\sigma_{t}=\left\{\begin{array}{ccc} E\varepsilon_{t}, & \; & {\varepsilon_{t}}^{p}<0.2\%,\\ K_{1}{\left({\varepsilon_{t}}^{p}\right)}^{n_{1}}, & \; & {\varepsilon_{t}}^{p}\geq 0.2\%, \end{array}\right.$$

where $\varepsilon_{t}$ is the true strain, the strength coefficient $K_{1}$ = 1215.7 MPa and the strain hardening exponent $n_{1}$ = 0.032, as displayed in Figure 5. The corresponding isotropic elastic parameters are the Young’s modulus *E* is 100 GPa and the Poisson’s ratio is 0.33. The model parameters of the extended GTN model are: the initial void volume fraction $f_{0}$ = 4 $\times \;10^{-6}$, $f_{c}$ = 0.5, $f_{F}$ = 0.6, κ = 3.0, $q_{1}$ = 1.5, $q_{2}$ = 1.0 and $q_{3}$ = 0.7. It should be mentioned that such a procedure for the parameters determination may not be unique; however, these determined material parameters can be used to qualitatively investigate the intergranular ductile fracture of the β titanium alloys influenced by both the grain boundary α layer and the grain interiors reasonably.

### 3.2. Damage Evolution in Tensile and Shear Loading

**Figure 6:**
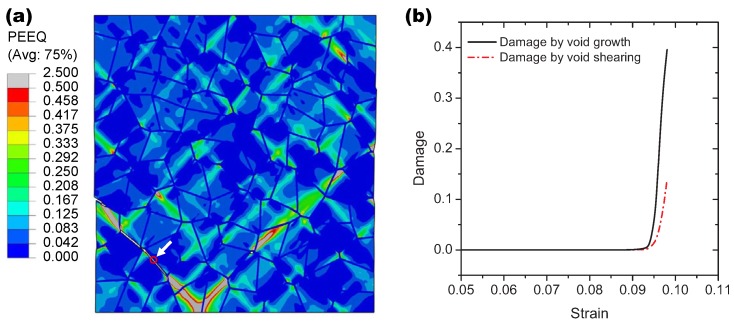
(**a**) the distribution of the equivalent plastic stain (PEEQ) within the grains under the uniaxial tension and (**b**) the corresponding damage evolution of one Gaussian point located in the red circle for both void growth and void shearing.

Accurate modeling of damage development is very important in capturing the macroscopic behavior of the lamellar Ti-5553 alloy since void growth and void shearing strongly affect the mechanical behavior of the alloy during deformation. For the uni-axial tension discussed above, the final distribution of the equivalent plastic stain within the grain interiors is displayed in Figure 6a. As shown by the graph, there are several positions within the grain interiors have the intense plastic strain concentrations. Actually, these positions also indicate the preferential plastic deformation along the adjacent α phase grain boundaries associated with the total applied stretch. Since the damage evolution is caused by both the plastic deformation and the stress states based on the current model, the intergranular cracks have been formed only in two places along the grain boundaries; meanwhile, the intergranular crack propagation is blocked by the high plastic strain localization ahead of the crack-tip. One of the Gaussian integration points located in the red circle indicated in Figure 6a is used to identify the damage evolution at the grain boundary. As shown in Figure 6b, the intergranular damage at the material point is caused by both void growth and void shearing during the tension process, where the damage growth rates for void growth and void shearing are calculated according to the first term and the second term of Equation (3), respectively.

**Figure 7:**
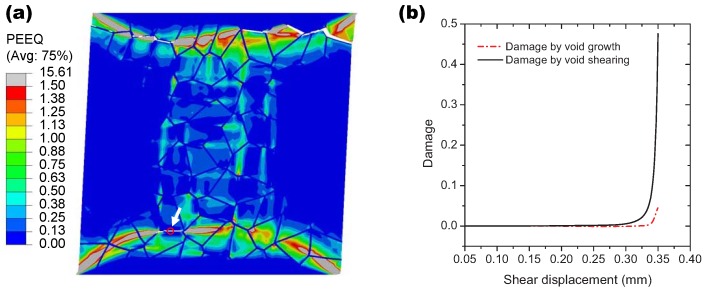
(**a**) the distribution of the equivalent plastic stain (PEEQ) within the grains under the shear loading and (**b**) the corresponding damage evolution of one Gaussian point located in the red circle for both void growth and void shearing.

**Figure 8:**
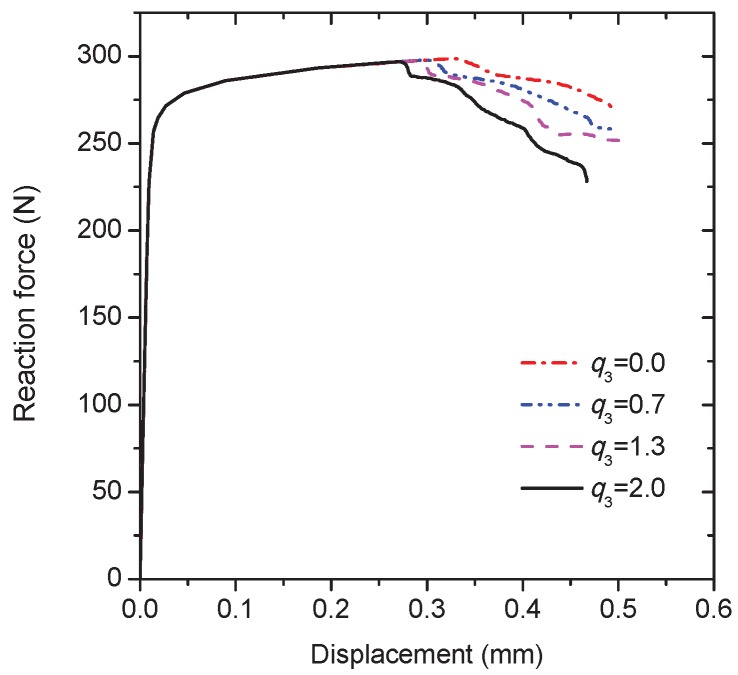
The reaction force versus the applied displacement curves of the RVE under the shear loading for different values of $q_{3}$.

In order to verify the application of the model under a shear dominated condition, a shear loading is performed on the RVE in the plane strain condition. All the top nodes are given the same shear displacements in the horizontal direction and one of the top nodes is constrained not to move in the vertical direction. The movements of both the horizontal and vertical direction are constrained for all the bottom nodes. The material constant $q_{3}$ = 2.0 and the rest of the model parameters are the same as those for the uni-axial tension simulation. The final distribution of the equivalent plastic stain within the grain interiors at the shear displacement of 0.46 mm is shown in Figure 7a. Although the loading is nearly antisymmetric, the intergranular crack paths are not antisymmetric due to the random grain geometry. These positions corresponding to the local plastic strain concentration indicate the preferential plastic deformation along the adjacent continuous α layer under the shear loading. The high plastic deformation within the grain interiors prevents the crack propagation through the grains, which is similar to that happened in the tension simulation. One of the Gaussian points inside the red circle as shown in Figure 7a is also used to capture the damage process of the grain boundary. Obviously, the void shearing mechanism dominates the damage development at the material point during deformation (see Figure 7b). The effect of $q_{3}$ is considered according to the reaction force against the shear displacement curve, as shown in Figure 8. Since $q_{3}$ is the acceleration factor of void shearing, the curve peaks at the lower value of displacement for the higher value of $q_{3}$. With the shear displacement, the reduction in force is also observed for $q_{3}$ = 0 due to the existence of the void growth mechanism. The above simulations reveal that the damage evolution associated with intergranular fracture can be captured reasonably by the current model.

## 4. Intergranular Crack Propagation

**Figure 9:**
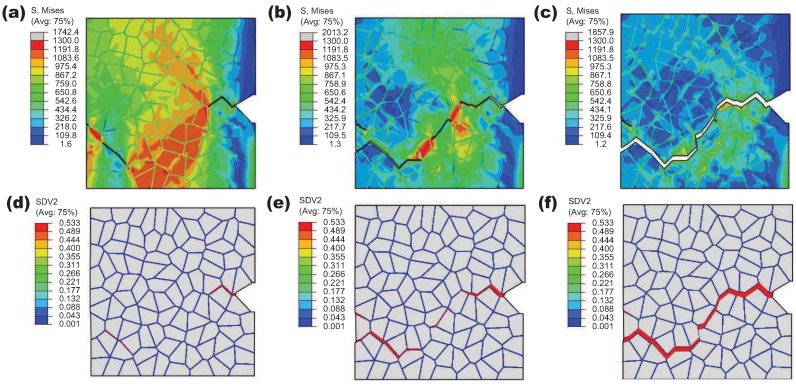
Contour maps during intergranular crack propagation: (**a**–**c**) von Mises stress; (**d**–**f**) damage evolution. The imposed displacement is (**a**,**d**) 0.04 mm; (**b**,**e**) 0.08 mm; (**c**,**f**) 0.3 mm.

After the model verification, the intergranular ductile crack propagation is simulated according to the determined material parameters. A notched specimen is created simply by removing one grain at the right boundary of the RVE, and the boundary conditions are the same as those for the uni-axial tension simulation. The calculation results for the distribution of the von Mises stress and the damage field evolution are displayed in Figure 9. The contour maps of the damage variable characterize crack propagation. There are several crack initiation sites, and two of them have the same positions as those in the uni-axial tension (see Figure 9a). With the further loading, the crack initiated from the notch-tip propagates into the bulk of material and merges with other cracks to form one main crack that crosses the polycrystalline aggregate. As shown in the von Mises maps, the high local stress concentrations happen ahead of the current crack-tips during crack propagation, and the local unloading occurs in the wake of the advancing crack-tips. Crack branching has also been observed at some triple points of the model; however, these crack branches stop to grow after propagating a very short distance (see Figure 9c). 

**Figure 10:**
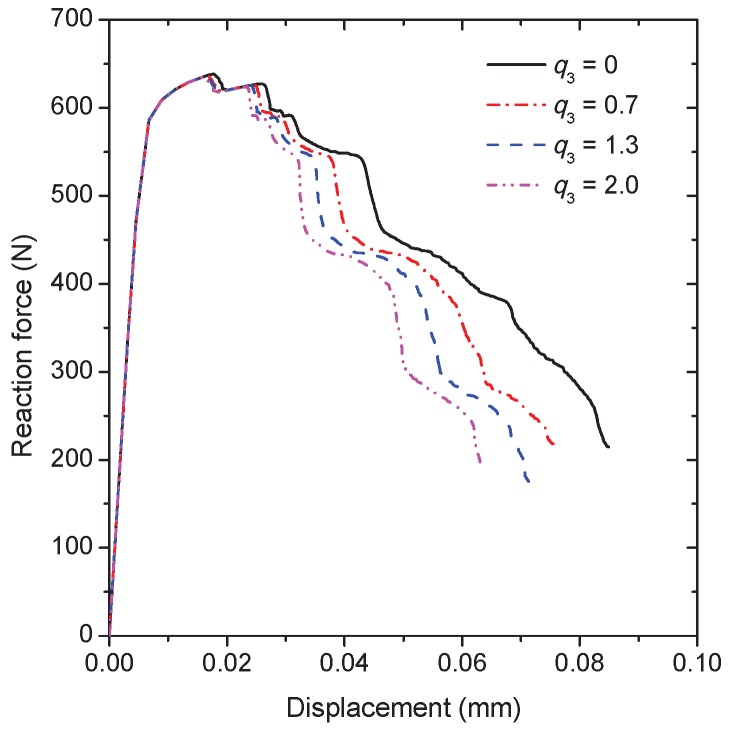
Computational results of the reaction force versus the applied displacement curves for different values of $q_{3}$.

Since changing the parameter $q_{3}$ results in a variation in the growth rate of voids, and a change of the strain at which fracture occurs [17], the effects of the void shearing mechanism on the intergranular crack propagation are considered by varying the value of $q_{3}$. The macroscopic load-displacement curves are compared for the different values of $q_{3}$ as plotted in Figure 10. As deformation progresses, the peak load is reached, and then the external load decreases due to the crack initiation at the notch-tip. However, there is no sudden drop of load after the crack initiation, instead there is a load plateau associated with the initial stage of crack propagation. The length of the load plateau for $q_{3}$ = 2.0 is shorter than other cases due to the faster void shearing process. Meanwhile, although the external load for $q_{3}$ = 2.0 drops more quickly during the subsequent deformation, the crack propagation path is exactly the same as other cases (see, e.g., Figure 9).

**Figure 11:**
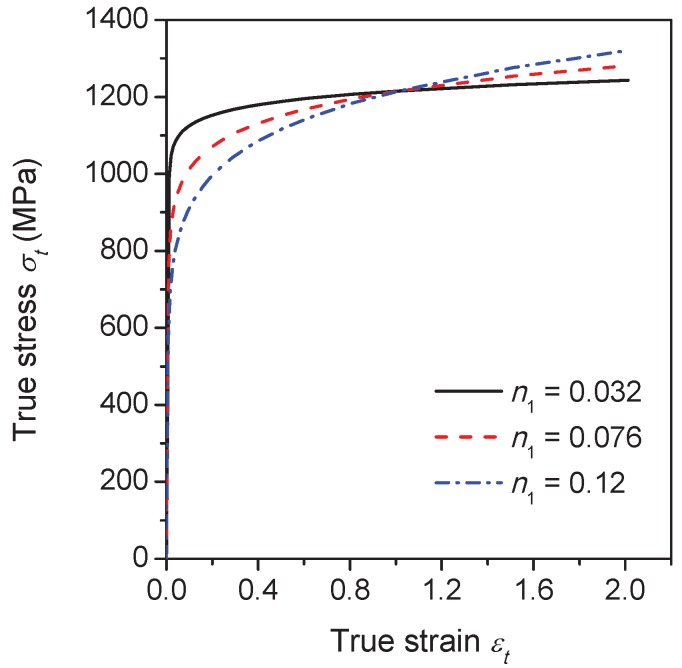
The true stress–strain curves of the grain interiors with the different strain hardening exponents.

**Figure 12:**
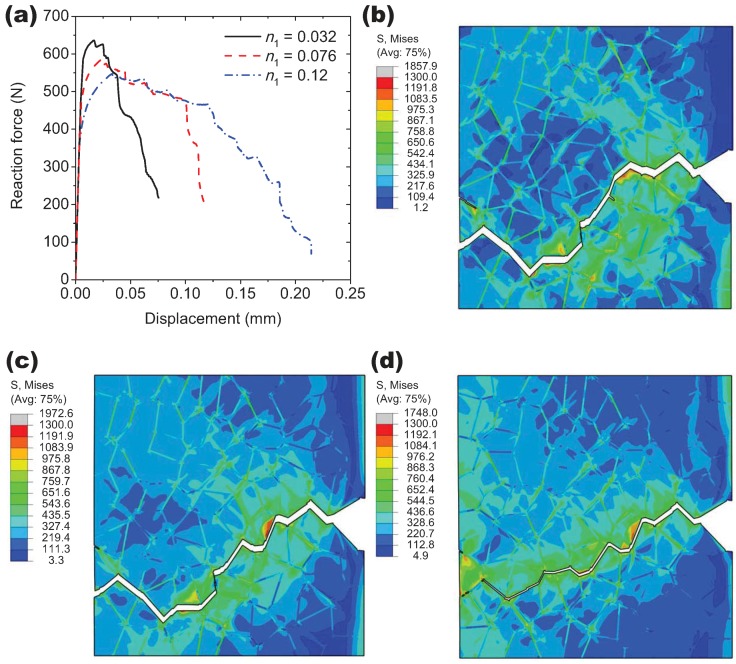
Fracture in a notched-tensile specimen: (**a**) reaction force versus displacement for the different strain hardening exponents; Contour maps in (**b**–**d**) show the von Mises stress in the grain interiors associated with the different crack propagation paths for (**b**) $n_{1}$ = 0.032, (**c**) $n_{1}$ = 0.076 and (**d**) $n_{1}$ = 0.12, respectively.

In order to explore the influence of the flow properties of the grain interiors on the intergranular crack propagation behavior, three different flow curves are considered by varying the strain hardening exponent $n_{1}$ in Equation (20), namely, $n_{1}$ is 0.032, 0.076 and 0.12, as illustrated in Figure 11. During calculations, the flow properties of the grain boundary α phase layer are fixed and $q_{3}$ = 0.7. The yield stress of the grain interiors ${\sigma_{y}}^{g}$ can be derived by substituting ${\varepsilon_{t}}^{p}$ = 0.2% into Equation (20). The ratio between ${\sigma_{y}}^{g}$ and the yield stress of the grain boundary α phase layer ${\sigma_{y}}^{\alpha }$ equals 1.3, 1.0 and 0.8 for each $n_{1}$ approximately. The simulation results for $n_{1}$ = 0.076 and 0.12 together with the former result of $n_{1}$ = 0.032 are compared in Figure 12. Based on the macroscopic load-displacement curves as shown in Figure 12a, the material softening occurs firstly at $n_{1}$ = 0.032 associated with the fastest crack initiation process, which indicates that the decrement of the strain hardening exponent of the grain interiors can promote the formation of the intergranular crack. It also can be seen that the peak load for $n_{1}$ = 0.032 is higher than the other two cases due to the higher ratio of ${\sigma_{y}}^{g}/{\sigma_{y}}^{\alpha }$. Since there is only one propagating crack that exists as shown in Figure 12d, the external load drops slower at $n_{1}$ = 0.12 in comparison with other two cases corresponding to the multiple crack propagation (see, e.g., Figure 9). 

**Figure 13:**
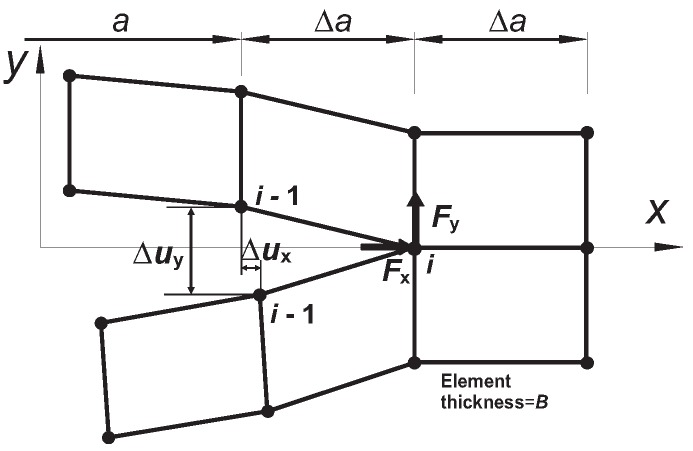
Illustration of the virtual crack closure technique (VCCT) through four nodal elements.

As mentioned, the intergranular fracture properties depend on the hardening ability of the grain interiors, the fracture toughness of Ti-5553 is estimated for the different strain hardening exponents. Since the plastic zone near the crack initiation site is constrained geometrically to the near region of grain boundary, the energy release rate *G*, which is valid in the small scale yielding, is adopted in this study. According to Irwin’s crack closure integral, the released energy *G* for a crack propagates from the length *a* to *a* + Δa is identical to the energy required to close the crack of length Δa. This idea has been realized according to the virtual crack closure technique (VCCT) [35] as the following:

(21) $$\left\{\begin{array}{c} G_{I}=\frac{1}{2B\Delta a}\left[F_{y}^{i}\left(a\right)\Delta u_{y}^{i-1}\left(a\right)\right],\\ G_{II}=\frac{1}{2B\Delta a}\left[F_{x}^{i}\left(a\right)\Delta u_{x}^{i-1}\left(a\right)\right], \end{array}\right.$$

where Δa is the crack propagation length and *B* is the thickness of the crack surface. As depicted in Figure 13, $F_{x}^{i}\left(a\right)$ and $F_{y}^{i}\left(a\right)$ are the nodal forces of node *i* at the crack-tip along *x* and *y* directions, $\Delta u_{x}^{i}\left(a\right)$ and $\Delta u_{y}^{i}\left(a\right)$ are the relative nodal displacements corresponding to the crack sliding and opening of the upper and lower crack surface at node *i*-1 along *x* and *y* directions, respectively. The total energy release rate *G* is the sum of $G_{I}$ associated with the opening mode and $G_{II}$ associated with the sliding mode. This technique has been implemented into ABAQUS according to the user defined element interface (UEL) [23] based on the algorithm introduced in Ref. [35].

**Figure 14:**
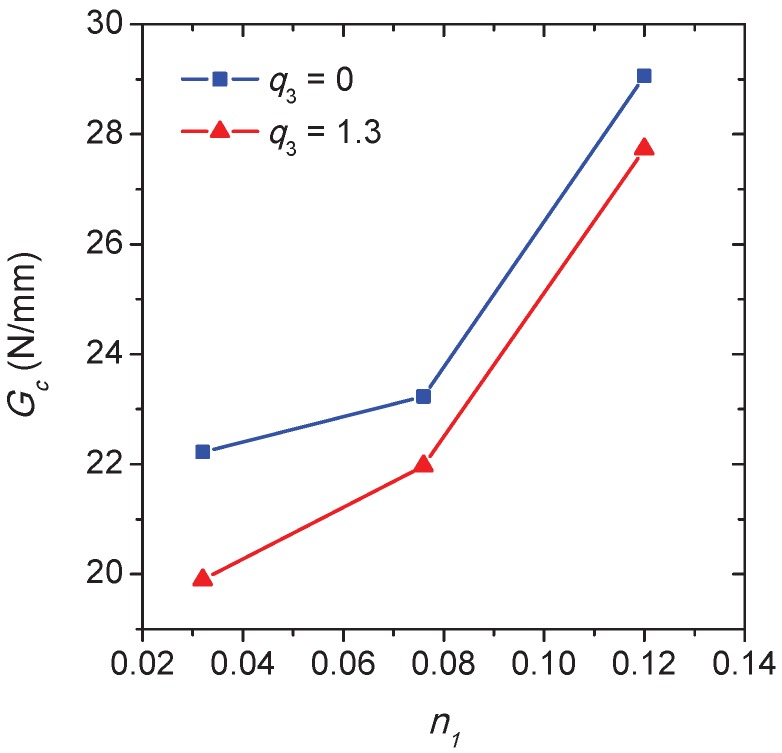
The critical energy release rate $G_{c}$ versus the strain hardening exponent $n_{1}$ curves for two different values of $q_{3}$.

Since fracture toughness is generally determined at the onset of crack propagation under the plane strain condition [36], the applied displacement corresponding to the crack length *a* = 0.02 mm is used to determine the critical energy release rate $G_{c}$. At this moment, there is only one crack that propagates for each $n_{1}$. The procedure for calculating $G_{c}$ is similar to that for the previous notch simulation, and the only difference is that the user elements are pre-defined along the crack propagation path according to the former numerical results. In order to examine the influence of the void shearing mechanism on fracture toughness, two different values of $q_{3}$ are considered to determine the released energy as shown in Figure 14, where *G* = $G_{I}$ + $G_{II}$. Since $G_{II}$ can be negligible compared with $G_{I}$ at *a* = 0.02 mm, the stress intensity factor for the opening mode can be derived based on $K_{I}$ = $\sqrt{E^{\prime }G}$ and $E^{\prime }$ = $E/(1-\nu^{2})$ for the plane strain condition. Assume that the Young’s modulus *E* = 120 GPa for the polycrystal according to the macroscopic stress–strain response as shown in Figure 5, and the Poisson’s ratio ν = 0.33, then the fracture toughness $K_{IC}$ = 60.7 MPa$\sqrt{m}$ for $q_{3}$ = 1.3 and $n_{1}$ = 0.12 approximately. This value is in agreement with the general fracture toughness found in the literature [1,37] for the Ti-5553 alloy with lamellar structure. In Figure 14, the variation of critical energy release rate $G_{c}$ as a function of the hardening exponent $n_{1}$ is plotted for two different values of $q_{3}$. In both cases, an increase of $n_{1}$ associated with a decrease of the ratio ${\sigma_{y}}^{g}/{\sigma_{y}}^{\alpha }$ can result in the higher fracture toughness. Meanwhile, the fracture toughness of Ti-5553 decreases with the increase of $q_{3}$ at a given $n_{1}$. Since the modifications of the yield stress or the strain hardening behavior of the grain interiors can be controlled by changing the size or the morphology of the intragranular α precipitate [37], the simulations indicate that the heat treatment associated with the improvement of the strain hardening can be performed to resist the intergranular fracture of the lamellar β alloys. 

**Figure 15:**
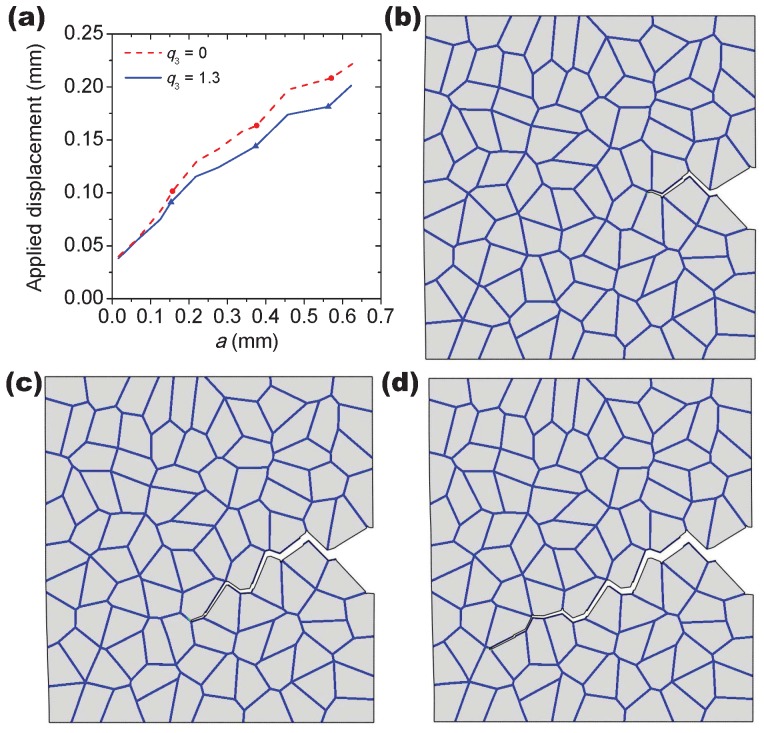
The crack propagation process in a notched-tensile specimen: (**a**) the applied displacement versus the crack length *a* curves at $n_{1}$ = 0.12. Contour plots in (**b**–**d**) show the moment corresponding to the different crack lengths indicated in (**a**).

**Figure 16:**
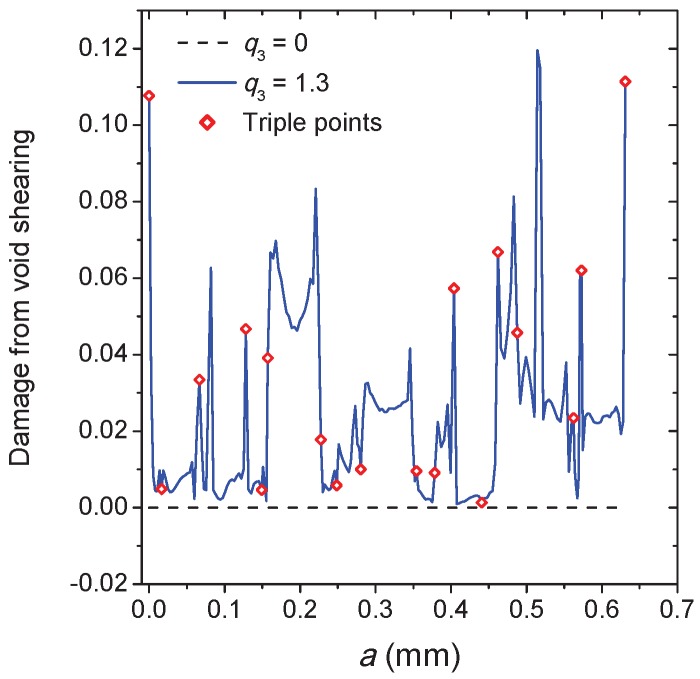
The damage caused by void shearing along the crack propagation path for two different values of $q_{3}$.

In Figure 15, the effect of void shearing on the crack propagation process is conducted for the hardening exponent $n_{1}$ = 0.12, unlike the other two cases of $n_{1}$ = 0.032 and 0.076, and only one predominant crack propagates in this case during deformation. As shown in Figure 14a, the applied displacement continuously increases with the crack length *a*, where the three points on each curve correspond to those moments of the crack propagation as displayed in Figure 15b–d. The sharp point on the curves indicates that the intergranular crack meets a triple point during propagation (see, e.g., Figure 15d). The damage accumulation caused by void shearing along the crack propagation path is shown in Figure 16. Depending on the stress field and the grain morphology, the damage associated with $q_{3}$ = 1.3 fluctuates significantly during crack propagation, and most of the peaks and valleys are located at the triple points. As expected, the presence of the void shearing mechanism strongly affects the intergranular crack propagation, and the increment of $q_{3}$ promotes the process of intergranular fracture. 

## 5. Conclusions

In this paper, the numerical simulations via the extended GTN model have been performed to study the intergranular ductile crack propagation of the β titanium alloy with lamellar structure. The individual phase properties of the grain boundary α phase layer and the hard grain interiors are determined according to the RVE numerically. Besides void growth, the void shearing of the grain boundary α phase can affect the macroscopic stress–strain response under both tension and shear dominated loading conditions significantly. An increase of the material constant $q_{3}$ associated with the acceleration of void shearing promotes the intergranular crack propagation process; however, the crack propagation path seems not to be influenced by changing $q_{3}$ solely. The existence of void shearing along the grain boundary α phase can be deleterious to the fracture toughness of the lamellar Ti-5553 alloy. The higher strain hardening exponent or the lower ratio of ${\sigma_{y}}^{g}/{\sigma_{y}}^{\alpha }$ results in the higher fracture toughness, and the crack propagation path can be affected by the hardening capacity of the grain interiors obviously. For engineering applications, the computational framework in this study can potentially be used as a guideline for the heat treatment process of the failure-resistant components made from the β titanium alloys with lamellar structure.

## Figures and Tables

**Table 1:** Chemical composition of the Ti-5553 alloy (wt %).

Ti	Al	*V*	Mo	Cr	Fe	*O*	*N*
Bal.	4.8	5.2	5.6	3.5	0.6	0.231	0.022
